# Polymer-specific nanoplastics trigger oxidative stress, inflammatory activation, and mitoepigenetic remodeling: *in vitro* and *in silico* evidence for MT-ND6-mediated mitochondrial complex I perturbation

**DOI:** 10.3389/ftox.2026.1916884

**Published:** 2026-08-31

**Authors:** Pradyumna Kumar Mishra, Aneha K. Rajan, Apoorva Chouksey, Vikas Gurjar, Ashwani Pathak, Aniket Aglawe, Ravi Prakash Tiwari, Debabrata Dash, Ram Kumar Nema, Rajnarayan Tiwari, Devojit Kumar Sarma, Rupesh K. Srivastava

**Affiliations:** 1 Division of Environmental Biotechnology, Genetics and Molecular Biology (EBGMB), ICMR-National Institute for Research in Environmental Health (NIREH), Bhopal, India; 2 Faculty of Medical Research, Academy of Scientific and Innovative Research (AcSIR), Ghaziabad, India; 3 Department of Biotechnology, All India Institute of Medical Sciences (AIIMS), New Delhi, India

**Keywords:** mitochondrial DNA, mitochondrial dysfunction, mitochondrial epigenetics, mitochondrial nuclear cross talk, mitochondrial redox signaling, mitochondrial remodeling

## Abstract

**Introduction:**

Recent studies have detected nanoplastics (NPs) in human tissues and biological fluids, raising concerns regarding their potential effects on cellular homeostasis. Nevertheless, their effects on mitochondrial regulation and mitoepigenetic processes remain poorly understood. This work aimed to study the polymer-specific effects of NPs, including polystyrene (PS), polypropylene (PP), and polyvinyl chloride (PVC), on mitochondrial function, mitochondrial homeostasis, oxidative stress generation, inflammatory response, and mitoepigenetics.

**Methods:**

Cell uptake of NPs was examined using flow cytometry and fluorescence microscopy. Analysis of mitochondrial membrane potential (Δψm), oxidative phosphorylation (OXPHOS), Mitochondrial reactive oxygen species (mtROS) generation, respiratory chain complexes, DNA damage repair enzymes, DNA methylation-related markers, inflammatory cytokines, and mitochondrial gene expression was performed at different time points after NP treatment. Moreover, docking analysis was performed to investigate the potential interactions between oxidized nanoplastic-derived oligomers and mitochondrial Complex I. The relationship between respiratory chain function and mtDNA expression was analyzed using regression.

**Results:**

The observed effects of the tested NPs included specific effects of each polymer type, such as increased mtROS production, reduced Δψm, induction of oxidative DNA damage, dysregulation of mitochondrial dynamics, and activation of inflammatory pathways. The stress response was characterized by downregulation of DNA methyltransferases, changes in methylation marker levels, and alterations in mitochondrial gene expression. Functional analysis identified Complex I as particularly vulnerable to the effects of NP treatment. The correlation study demonstrated the coordinated regulation of stress-response mediators in mitochondria, namely, DRP1, OMA1, DELE1, and MT-ND6. Meanwhile, the regression analysis showed a correlation between MT-ND6 expression and Complex I activity.

**Discussion:**

Collectively, these findings suggest that environmentally relevant NPs elicit polymer-dependent mitochondrial stress responses, mitoepigenetic remodeling, and inflammatory activation.

## 1 Introduction

Plastics, which are used extensively in many industries, break down into Microplastics (MPs) (<5 mm) and Nanoplastics (NPs) (<1,000 nm) (WHO, 2024). Because of their small size, chemical stability, and high surface reactivity, NPs such as PS, PP, and PVC persist in the environment and pose risks to ecological integrity and human health through multiple exposure routes (Enfri et al., 2020; Nazeer et al., 2024). These particles have been found in a variety of environmental compartments, such as the air (Dris et al., 2015), water, soil, and food sources. They have also been shown to be systemically distributed in people by ingestion, inhalation, and skin contact. Each of these routes has distinct toxicokinetic consequences, but NPs consistently cross biological barriers and become systemically distributed throughout the body regardless of the route of entry (Prata, 2018; Wang et al., 2024; Ratre et al., 2024). Upon exposure, the NPs develop a protein-lipid corona of high biological complexity and reactivity, which plays a crucial role in defining their biological identity and toxicity profile (Lima et al., 2020). Following internalization, the NPs encounter the plasma membrane (Lesniak et al., 2013) and enter via passive diffusion and active endocytosis (Fiorentino et al., 2014).

Emerging evidence suggests that the epigenetic regulation of mitochondria by redox states, metabolites, and non-coding RNAs (ncRNAs) is a fragile yet understudied target of external stressors (Lees et al., 2023; Rajan et al., 2025). NPs have been shown to disrupt mitochondrial gene control by perturbing redox homeostasis and metabolism, thereby influencing mitochondrial DNA (mtDNA)- encoded transcriptional programs independently of overt structural mitochondrial damage (Tao et al., 2024; Mondal et al., 2025). This could be due to the inhibitory action of NPs on the Electron Transport Chain (ETC), the generation of reactive oxygen species (ROS), and the disturbed calcium balance, resulting in persistent bioenergetic dysfunction (Bu et al., 2024; Chulkov et al., 2025). In animal models, these phenomena have been observed to result in inflammatory and immunotoxic/neurotoxic effects (Wang et al., 2021; Prosperi et al., 2025), whereas *in vitro* experiments with human cells have shown that these materials can be effectively taken up by cells (Sendra et al., 2020; Xu et al., 2021). Peripheral blood mononuclear cells (PBMCs) were chosen as an *ex vivo* system because of their high mitochondrial content and sensitivity to various environmental factors, facilitating the investigation of mitochondria-related mechanisms of NP toxicity, as well as the immune response and accompanying epigenetic changes (Mishra et al., 2022a; Soni et al., 2025; Garrafa et al., 2023; Hernández et al., 2025). PS, PP, and PVC were selected for comparative analysis due to their environmental prevalence and frequent detection in air, water, food, and human tissues (Visileanu et al., 2025). These polymers have also been detected in human tissues like lungs, blood, placenta, and brains (Thompson et al., 2024), where they have been associated with ROS generation, ETC dysfunction, loss of *ΔΨ*m, ATP depletion, and mtDNA damage, which have been associated with activation of inflammatory signaling pathways and increased production of pro-inflammatory cytokines (Bhargava et al., 2019a).

In this study, we examined the effects of PS, PP, and PVC NPs on mitochondrial function and epigenetic regulation in PBMCs to contribute to a broader understanding of how NP exposure may influence immune and metabolic processes and their possible role in long-term health risks. Attention was given to the mtDNA-encoded ND6 gene, which encodes a hydrophobic transmembrane subunit located within the proximal membrane arm of Complex I, where it is thought to contribute to ubiquinone reduction and proton translocation across the inner mitochondrial membrane (Vartak et al., 2015). Changes in ND6 expression may influence Complex I assembly, contribute to electron leakage, and enhance ROS buildup, although the extent to which such transcriptional changes have functional repercussions is unknown (Yin et al., 2021). Despite its central role in Complex I function, the potential involvement of ND6 in nanoplastic-induced mitochondrial stress remains poorly understood. To identify potential physical interaction sites between the oligomers of NPs and Complex I, a molecular docking simulation was conducted at specific electron-transfer sites to discover possible binding configurations that may affect enzyme activity. Additionally, supervised regression analysis was conducted to investigate the relationship between ND6 expression values (determined by PCR) and Complex I activity.

## 2 Materials and methods

### 2.1 Blood sample collection and lymphocyte isolation

Peripheral blood was withdrawn from 30 healthy volunteers (15 males and 15 females), aged 20–35 years, by venipuncture. Written consent was obtained from everyone, with permission from the Institutional Ethics Committee (IEC). Those subjects having confounding factors like smokers, alcoholics, or any exposure were not included in the experiment to prevent any interference in the cellular response. The samples were sent to the laboratory in an ice box and analyzed. Separation of PBMCs was performed by density gradient centrifugation using HiSep LSM 1077 (HiMedia Laboratories, Mumbai, India). Cells, after separation, were washed thoroughly with 1× Phosphate-buffered saline (PBS) (HiMedia Laboratories, Mumbai, India) to remove any trace of plasma and other contaminants and then suspended in PBS (Bhargava et al., 2015; Mishra et al., 2021; Suresh et al., 2017).

### 2.2 Preparation, characterization, and uptake of nanoplastics

Polymers PS, PP, and PVC were supplied by Sigma-Aldrich (Merck, United States). The first step is to dissolve polymers in solvents such as DMSO, xylene, and chloroform. The solution was then stirred using a magnetic stirrer at room temperature for 2–3 h. The second step is sonication to prevent particle agglomeration. Next, the suspensions were filtered through membrane filters to obtain smaller plastic particles prior to characterization (Gurjar et al., 2024). The physicochemical properties, such as hydrodynamic particle size distribution and zeta potential, of the prepared nanoparticles were measured using a Zetasizer Nano ZS (Malvern Panalytical, United Kingdom). The hydrodynamic particle size distribution was measured using dynamic light scattering (DLS), while the zeta potential of the nanoparticles was determined by the electrophoretic light scattering (ELS) technique (Arribas et al., 2024; Bhargava et al., 2019b). Cellular uptake of the NPs was first assessed using commercially available fluorescently labeled polystyrene nanoplastics (PS-NPs) (FluoSpheres™ carboxylate modified microspheres; Thermo Fisher Scientific, United States) as a model system for visualization of nanoparticle uptake. Post-incubation, the cells were extensively washed with PBS to remove extracellular nanoparticles. Intracellular localization of the PS-NPs was visualized by fluorescence microscopy (IX83, Olympus Corporation, Japan), and quantitative cellular uptake was assessed by measuring fluorescence intensity in the gated lymphocyte population using an Attune™ NxT flow cytometer (Thermo Fisher Scientific, United States). Additionally, comparative cellular uptake data for PS, PP, and PVC NPs obtained under identical experimental conditions are provided to complement the biological findings presented in this study (Supplementary Table S1). All subsequent mechanistic investigations, including oxidative stress, mitochondrial bioenergetics, mtDNA methylation, gene expression, inflammatory cytokine profiling, and mitochondrial respiratory chain analyses, were performed using the corresponding unlabeled PS, PP, and PVC NPs (Bunkar et al., 2020).

### 2.3 Treatment and incubation of lymphocytes with nanoplastics

In the *ex vivo* exposure experiment, PBMCs obtained via density gradient centrifugation were separately exposed to PS, PP, and PVC NPs at a concentration of 1 μg/mL, prepared from their respective stock suspensions, while non-exposed PBMCs served as the control group. This dose (1 μg/mL) was chosen based on previous *in vitro* studies of NPs that showed measurable biological responses in PBMCs and other mammalian cells without overt cytotoxicity. This dose was chosen to represent an environmentally relevant exposure level, as observed in recent human biomonitoring studies, allowing the study of molecular and cellular reactions under biologically relevant conditions. Approximately 1 × 10^6^ PBMCs were added to 2.5 mL of HiKaryoXL™ RPMI medium (HiMedia Laboratories, Mumbai, India). PHA-P acted as a mitogenic inducer, enhancing lymphocytes’ proliferative capacity. The PBMC cultures were incubated at 37 °C in a high-humidity, CO_2_-enriched incubator. Subsequently, PBMC cultures were treated with PS, PP, and PVC NPs after being activated for 0, 6, 24, and 48 h. Unexposed PBMC cultures were used as controls in parallel with treated samples at every time point (Adamiak et al., 2025; Mishra et al., 2022b).

### 2.4 Analysis of cellular oxidative damage and intracellular stress

Isolation of DNA from PBMCs was performed according to the manufacturer’s instructions with the PureLink™ Genomic DNA Mini Kit (Invitrogen, Thermo Fisher Scientific, Waltham, MA, United States). For the measurement of DNA damage, levels of oxidized purines were quantified by measuring 8-oxo-guanine formed during the formamidopyrimidine DNA glycosylase (fpg)-catalyzed reaction. ROS generation was assessed by oxidation of CM-H2DCFDA (Invitrogen, Thermo Fisher Scientific, Waltham, MA, United States) to DCF, as measured by fluorescence. Alterations in Δψm were evaluated, and induction of apoptosis and necrosis was assessed using CellEvent™ Caspase-3/7 Green Detection Reagent (Invitrogen, Thermo Fisher Scientific, Waltham, MA, United States) along with SYTOX™ AADvanced™ reagent (Bunkar et al., 2020).

### 2.5 Gene expression analysis by quantitative real-time PCR

Total RNA isolation was performed using TRIzol™ Reagent (Life Technologies, Thermo Fisher Scientific, Waltham, MA, United States) according to the company’s instructions. RNA concentration and purity were measured spectrophotometrically before cDNA synthesis. qRT-PCR analysis was performed using the InstaQ-96™ Real-Time PCR System (HiMedia Laboratories, Mumbai, India). RNA expression profiling of markers of mitochondrial dynamics, including fission (*DRP1*) and fusion (*MFN1*), and of mitochondria-encoded OXPHOS transcripts, including *MT-ATP6*, *MT-COX1*, and *MT-ND6*, was performed. Furthermore, quantitative gene expression analysis of genes involved in mitochondrial stress-response signaling pathways (including *OMA1* and *DELE1*), DNA repair processes (including *APE1* and *OGG1*), and epigenetic machinery (including *DNMT1*, *DNMT3a*, and *DNMT3b*) was carried out. Expression analysis was carried out relative to the endogenous reference gene *GAPDH* using the 2^−ΔΔCt^ method (Soni et al., 2025).

### 2.6 Quantification of mitochondrial DNA methylation

Methylation of mtDNA was done through bisulfite conversion followed by methylation-specific PCR (MSP). Genomic DNA was extracted from both NP-treated and non-treated PBMCs. Prior to bisulfite modification of total DNA, it was digested with BamHI at 37 °C for 4 h. The bisulfite conversion changes unmethylated cytosine to uracil while the methylated cytosine remains unaffected. DNA samples were treated with bisulfite modification buffer at 98 °C for 10 min, then at 60 °C for 150 min. Following the treatment, the DNA samples were desulfonated, purified, and eluted as per the manufacturer’s instructions. MSP analysis was then conducted using primer pairs for selected mtDNA targets, including D-loop1, D-loop2, 12S rRNA, and 16S rRNA. PCR amplification reactions were done using the Insta Q-96™ Real-Time PCR System (HiMedia Laboratories, Mumbai, India). At the end of amplification, Ct values were measured, and relative methylation levels of each mitochondrial target region were estimated by comparing Ct values between methylated and unmethylated primer sets across the experimental groups (Mishra et al., 2022a).

### 2.7 Gene expression analysis of miRNA and its target genes

MiRNAs such as miR-21, miR-34a, and miR-155, which have been correlated with inflammation, apoptosis, and oxidative stress responses, were analyzed by quantitative real-time PCR (qRT-PCR). After NP treatment, PBMCs were harvested, and total miRNA was extracted using the PureLink™ miRNA Isolation Kit (Thermo Fisher Scientific, Waltham, MA, United States) according to the manufacturer’s protocol. Poly (A) tailing of miRNAs isolated from PBMCs at their 3′ ends was achieved using poly (A) polymerase. Reverse transcription of miRNAs was done using an oligo (dT) primer and reverse transcriptase, under preoptimized reaction conditions. Quantitative analysis of the synthesized cDNAs was performed by qRT-PCR using primers for miR-21, miR-34a, and miR-155. The U6 small nuclear RNA served as the internal control for miRNAs. Expression analysis of microRNAs (miRNAs) and their target genes, the expression levels of *PTEN*, *FOXO3*, and *BCL2*, which are targets of miR-21, miR-34a, and miR-155, respectively, were quantified by qRT-PCR. Relative mRNA expression was calculated using GAPDH as the endogenous control. Relative expression levels were calculated using the 2^^−ΔΔCt^ method (Mishra et al., 2021).

### 2.8 Evaluation of inflammatory cytokine levels

To determine inflammation induced by NP exposure, the levels of Interleukin 6 (IL-6), Interleukin 8 (IL-8), and Tumor necrosis factor-α (TNF-α) were quantified using the Human GENLISA™ ELISA kit (KRISHGEN BioSystems, Cerritos, CA, United States) as described by the manufacturer. Supernatant samples obtained at selected time points of NP exposure were measured for cytokine levels. The measurement was performed using a Spark® multimode microplate reader (Tecan, Switzerland), and cytokine concentrations were calculated from their standard curves (Mishra et al., 2022b).

### 2.9 Assessment of mitochondrial ETC complex activity

Activities of mitochondrial ETC complexes were examined after treatment with PS, PP, and PVC NPs. PBMCs obtained at different incubation times (0, 6, 24, and 48 h) were further processed according to the manufacturer’s protocol using MitoCheck Complex Activity Assay Kits (Cayman Chemical, Ann Arbor, MI, United States). Activities of mitochondrial respiratory chain complexes were estimated spectrophotometrically using a Spark® multimode microplate reader (Tecan, Switzerland). Relative enzyme activities were calculated from absorbance values obtained in the experiment (Soni et al., 2025).

### 2.10 *In silico* docking studies

*In vitro* molecular docking studies were conducted to elucidate the mechanisms of interaction between oxidized nanoplastic-derived oligomers and mitochondrial complex I. The crystal structure of human mitochondrial complex I (PDB code: 5XTD) was retrieved from the Protein Data Bank (PDB) and prepared using the Protein Preparation Wizard in the Schrödinger Maestro Suite. This included the introduction of hydrogen atoms, calculation of bond order, optimization of protonation states, enhancement of hydrogen bonding, and minimization of energy while applying restraints using the OPLS4 force field. Solvent molecules located far from the functionally relevant sites were removed, and grids were created for the proteins at selected catalytic and cofactor-binding sites. This study has been conducted to optimize sixteen representative oxidized oligomeric fragments of PS, PP, and PVC bearing functional groups such as -OH, -COOH, and -CHO attached at the α- or ω-position. Oligomeric representatives were chosen because the bulky nature and conformational variability of polymer chains do not meet the requirements of standard protein-ligand docking techniques. Additionally, due to environmental oxidation of plastics, low-molecular-weight oxidized polymer fragments, such as monomers and oligomers, appear and serve as realistic chemical compounds for studying their interactions with biological molecules. Therefore, oligomeric representatives were used as surrogates to identify potential binding sites between oxidized nanoplastic fragments and mitochondrial complex I. The optimization was done by LigPrep software at a physiological pH of 7.4 ± 0.2. The molecular docking study was performed using the Glide program in extra precision mode, and the ten best-binding orientations were obtained for each ligand. The docking poses were minimized, and the binding affinity ranking for the obtained docking complexes was based on GlideScore (kcal/mol); the more negative the scores, the higher the predicted binding affinity. Protein-ligand interactions were visualized and analyzed using the Maestro Pose Viewer software for hydrogen bonding, electrostatic interactions, hydrophobic interactions, and π-π stacking interactions. The docking analysis was expected to provide a computational approach to studying potential binding sites for oxidized nanoplastic-derived oligomers at Complex I of mitochondria; it does not constitute evidence for the actual existence of such molecular interactions (Soni et al., 2026).

### 2.11 Regression-based analysis of the association between complex I activity and *MT-ND6* expression

To elucidate a possible link between Complex I activity and *MT-ND6* gene expression, a regression-based data analysis method was used. Enzyme activity data from enzymatic assays served as predictor variables, while expression data from qRT-PCR served as response variables. Both types of variables were normalized to ensure comparable results. All available data across treatment groups and observation times were randomly split into training (80%) and test (20%) datasets. Random Forest regression with multi-output was applied to determine whether there was a pattern of dependence between Complex I activity and *MT-ND6* gene expression levels. The quality of the resulting model was assessed using the coefficient of determination (R^2^) and the mean squared error (MSE) on the test set. Residuals and plots of predicted vs. observed variables were also considered. It should be noted that the aim of this analysis was simply to measure the strength of dependence and thus should not be viewed as proof of a causal relationship between the predictor and response variables (Kaur et al., 2025).

### 2.12 Statistical analysis

Statistical analysis was performed using Python. Data processing and organization were performed using the Pandas and NumPy libraries, while graphics were plotted using the Matplotlib and Seaborn libraries. Data are presented as means ± standard error of the mean (SEM). To check the data distribution for normality, the Shapiro-Wilk test was used. Multiple comparisons among experimental groups were performed using one- or two-way ANOVA, followed by Tukey’s *post hoc* test. In cases where the normality assumptions did not apply, the data were tested using the Kruskal-Wallis test, followed by Dunn’s multiple comparisons test. The statistical significance threshold was set at p < 0.05. Pearson correlation was used to determine correlation coefficients between biological variables. The correlation matrix was generated to identify possible associations among mitochondrial, epigenetic, inflammatory, and metabolic factors. In case of regression analysis, data from different groups (exposure groups: PS, PP, PVC; different exposure times: 0, 6, 24, 48 h; control samples) were pooled together, split randomly into a training set (80% of the total data) and an independent test set (20% of the total data), and then analyzed as described before (Kaur et al., 2025).

## 3 Results

### 3.1 Characterization and cellular uptake of NPs

**FIGURE 1:**
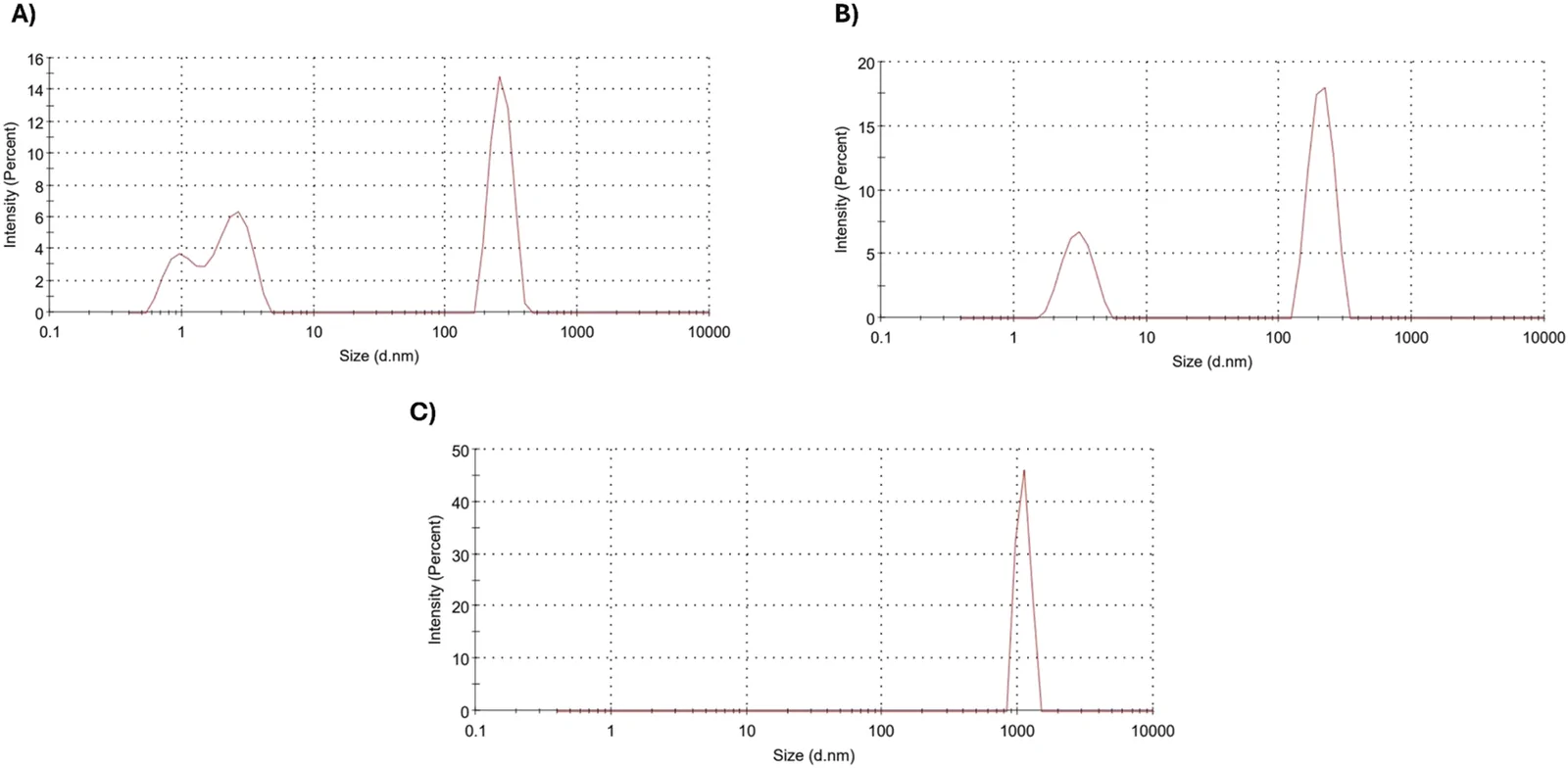
Particle size distribution of polymers measured by DLS. The hydrodynamic size distributions of **(A)** polystyrene (PS), **(B)** polypropylene (PP), and **(C)** polyvinyl chloride (PVC) NPs in aqueous suspension.

**FIGURE 2:**
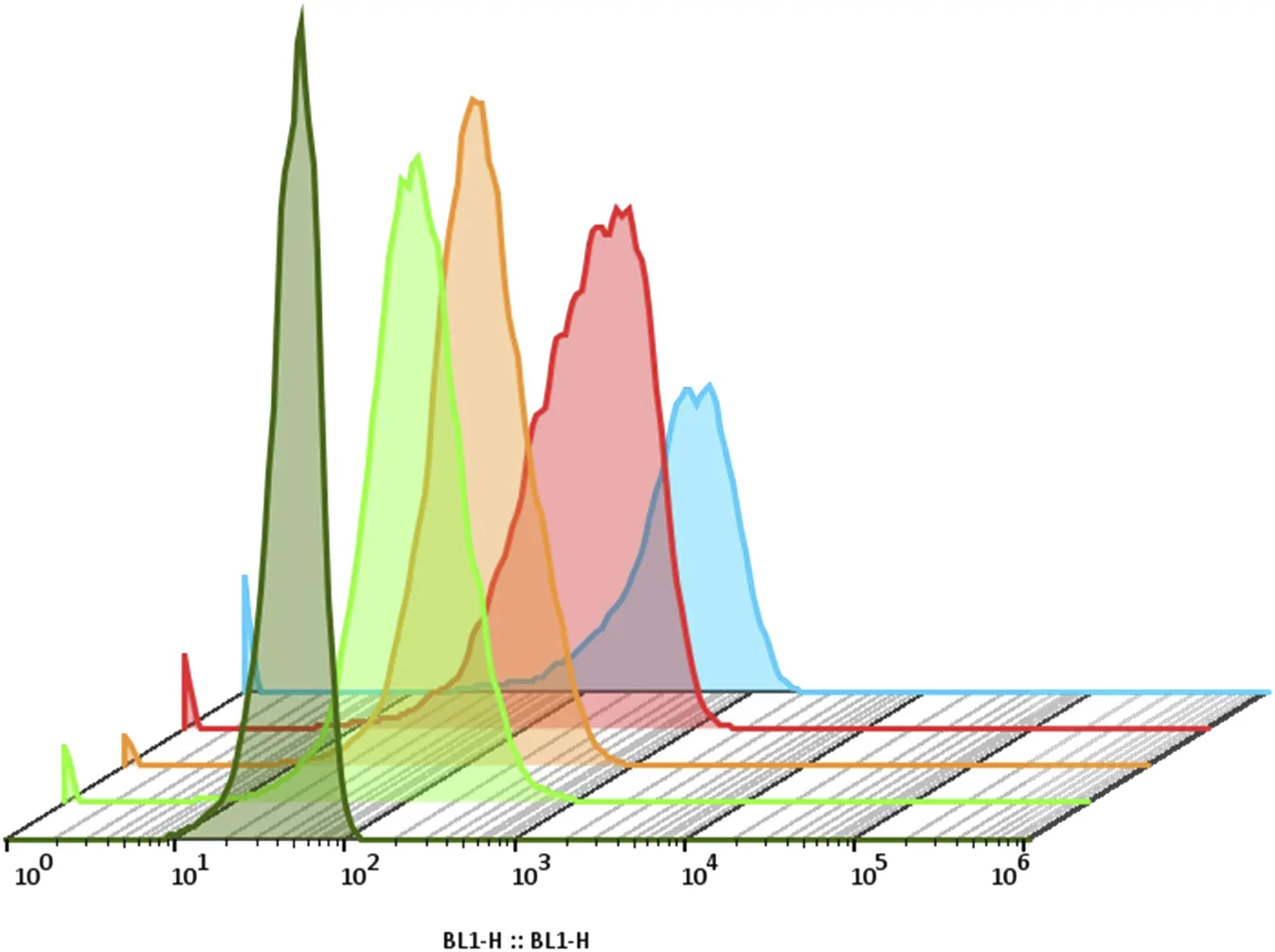
Flow cytometric analysis of time-dependent PS uptake by lymphocytic cells. Overlayed staggered histograms show the fluorescence intensity (BL1-H) of lymphocytic cells following exposure to PS at various time points. The control is shown in black, and the time points (0, 30, 60, and 120 min) are shown in green, orange, red, and blue, respectively. A rightward shift in fluorescence intensity was seen over time, indicating increased uptake of PS particles.

The physicochemical characterization of the prepared NPs was performed using DLS analysis and zeta potential determination in aqueous suspension. From the DLS, a difference in hydrodynamic size distribution was observed among the three polymer types. PS nanoparticles possessed the smallest average hydrodynamic diameter with the value of (87.4 nm, Figure 1A), followed by the PP nanoparticles (148.4 nm, Figure 1B). PVC nanoparticles had the largest average hydrodynamic diameter (227.4 nm, Figure 1C) with a significant intensity peak representing the presence of most of the particles. For zeta potential determination, a nearly neutral surface charge was observed for all types of NPs, with values of −3.39 mV (PS), −0.457 mV (PP), and −0.317 mV (PVC). The initial cellular uptake of the PS NPs was assessed using DIC-fluorescence microscopy (Supplementary Figure S1) to confirm their internalization by PBMCs. ImageJ-based particle analysis of representative microscopy images showed a progressive increase in intracellular NP uptake as exposure time increased. No particles were detected at 0 min, whereas particle counts increased at 1, 3, and 6 h for all NPs type. At 6 h, intracellular particle counts were 823 ± 26 for PP, 680 ± 25 for PVC, and 539 ± 19 for PS, indicating greater apparent cellular accumulation of PP than of PVC and PS under identical imaging and analysis conditions (Supplementary Table S1). As expected, control cells presented a uniform cellular morphology without a fluorescence signal. In contrast, after treatment of cells with PS nanoparticles labeled with a fluorescent dye, discrete fluorescent puncta within the cell body became visible at 1 h. With longer incubation periods, fluorescence intensity increased. By 3 h, numerous fluorescent puncta were seen scattered across the cytoplasm. During the 6 h exposure period, bright fluorescent puncta were detected, indicating substantial uptake of PS nanoparticles by the cells. In addition, to quantify PS nanoparticle cellular uptake, flow cytometry was used to measure fluorescence intensity (BL1-H channel). The control group of cells showed no change in fluorescence intensity. There was a gradual right shift of the curves with prolonged exposure times from the control group of cells (Figure 2). The early exposure (30 min) showed a slight increase in fluorescence intensity from that of the control group, while exposure for 60 min resulted in an obvious increase in mean fluorescence intensity. The highest fluorescence intensity was recorded after 120 min of exposure to PS nanoparticles.

### 3.2 Oxidative damage and intracellular stress

**FIGURE 3:**
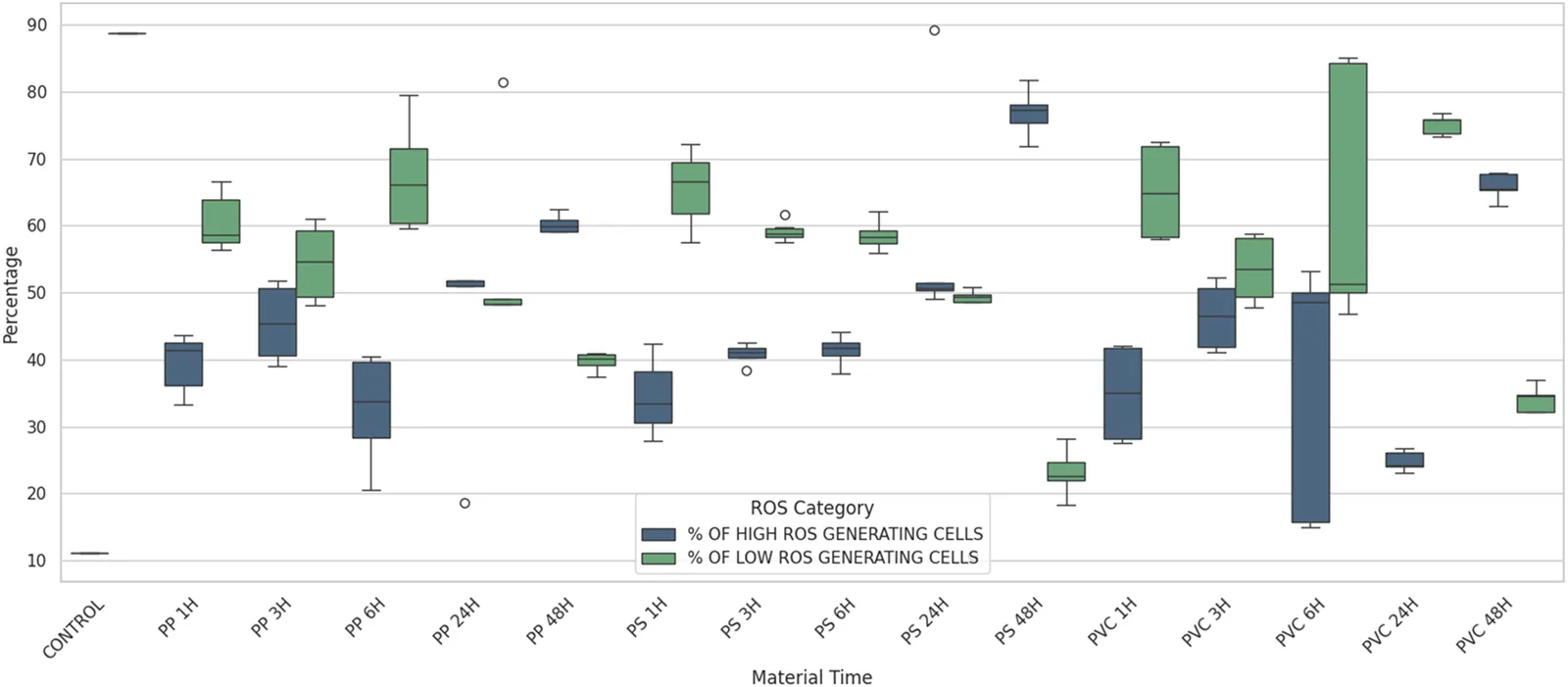
Box plot of the percent of ROS-positive cells. The Y-axis represents the percentage of ROS-producing cells, while the X-axis indicates the experimental groups. The box contains the interquartile range of the data, with the median indicated inside the box.

The findings of the FPG Enzyme Test showed that the level of FAPY adducts in DNA from all NP-exposed groups was higher than in unexposed controls (see Supplementary Figure S2). It has been observed that FAPY expression in the PP-exposed group first appeared after 6 h of exposure, while for PS and PVC, it happened later. However, 24 h after treatment, there was an overall decline in FAPY expression across all groups; FAPY expression remained positive in PP- and PVC-treated samples. A further decrease in FAPY expression was observed after 48 h, particularly in PVC samples. The concentration-dependent increase in intracellular ROS levels in all three types of NP-treated cells, as determined by flow cytometry with CM-H2DCFDA, was observed (Figure 3). PP-treated cells exhibited the most significant response in ROS generation. PS and PVC showed an early peak, followed by a partial decline, then a second peak at later times. CellROX™ Deep Red-stained mitochondria showed a similar trend (Supplementary Figure S3), with the peak occurring at 6 h across all group s and the elevation maintained through 48 h in PP-treated cells. Activity of caspase-3/7 reflected a dose-dependent cell death mechanism (Supplementary Figure S4), with PP showing persistent induction of necrosis consistent with the ROS production pattern. ΔΨm decreased significantly in all groups (Supplementary Figure S5). Thus, only 30%–37% of cells retained mitochondrial membrane polarization at 6 h. The percentage of highly polarized cells decreased further after 24 h, especially in the PVC-treated group, where only ∼13–17% of cells remained.

### 3.3 Gene expression analysis

Gene expression related to mitochondrial dynamics, OXPHOS, DNA damage response/repair, integrated stress response, and epigenetics was altered by exposure to PS-, PP-, and PVC-NPs. *DRP1* expression levels were significantly increased in all NP-treated samples compared with the control (Supplementary Figure S6). PS-exposed cells exhibited maximum induction of expression levels at 6h, followed by a decrease in later exposure periods, while PP- and PVC-exposed cells expressed moderately higher levels of expression throughout the exposure periods. Expression levels of the *MFN1* gene were increased in all three samples; however, the magnitude and timing of induction differed by polymer type. The effects of NPs on mitochondrial genome-encoded genes for OXPHOS (*MT-ATP6*, *MT-COX1*, and *MT-ND6*) were assessed to evaluate transcriptional responses in mitochondrial bioenergetics (Supplementary Figure S7). Induction of *MT-ATP6* expression was progressive in PS-exposed cells, reaching a maximum after 48 h of exposure; however, PP- and PVC-exposed cells showed a relatively smaller increase in *MT-ATP6* expression. Expression levels of *MT-COX1* were similarly induced in PS-exposed cells and PVC-exposed cells. Similar results were observed for the *MT-ND6* gene, with the highest induction of expression in PS-exposed cells, followed by PP- and PVC-exposed cells.

Gene expression patterns that depended on polymers were seen for BER-associated genes (Supplementary Figure S8). The *OGG1* gene expression level increased markedly after 24 h of PS treatment, whereas PP and PVC treatments showed a moderate increase. *APE1* gene expression was higher in PVC-treated samples after 48 h. Gene expression levels of mitochondrial stress-associated factors, such as *DELE1* and *OMA1*, were increased across all groups, indicating that these factors were activated by NP exposure (Supplementary Figure S9). Analysis of DNA methyltransferase genes showed a pattern of increasing suppression of gene expression after NP exposure (Supplementary Figure S10). After 48 h of treatment, *DNMT1* showed a significant reduction in gene expression, from a medium level at 6 h to a low level. DNMT3a also showed constant suppression, whereas *DNMT3b* showed polymer-dependent fluctuations that decreased after 48 h. The greatest reduction in DNA methyltransferase expression was observed in PP-treated cells, followed by PVC- and PS-treated cells (p < 0.01).

**FIGURE 4:**
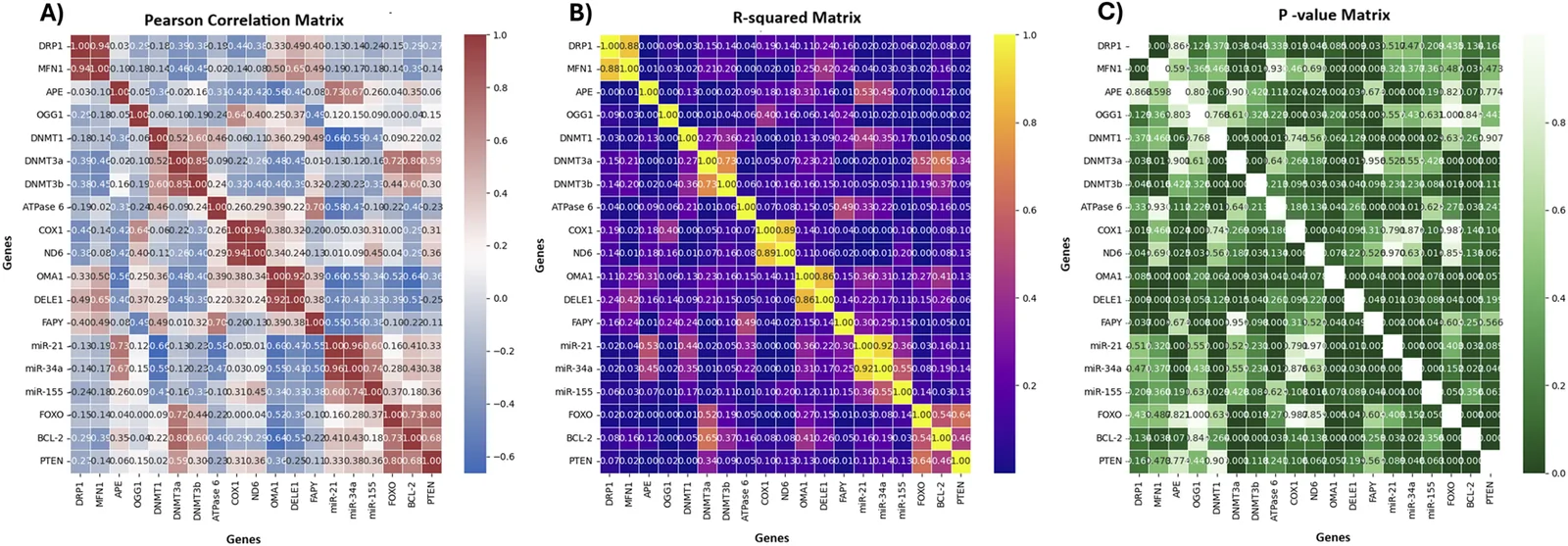
Correlation analysis of mitochondrial, epigenetic, and regulatory genes. Heatmaps showing relationships between the analyzed genes. **(A)** Pearson correlation coefficients, **(B)** Coefficients of determination (R^2^), **(C)** Color scales represent the corresponding p-values.

A Pearson correlation study yielded significant relationships amongst genes responsible for mitochondrial dynamics, OXPHOS, DNA repair, epigenetic modifications, and miRNAs associated with mitochondria (Figure 4). For instance, *DRP1* showed a high positive correlation with *OMA1* (r = 0.96, R^2^ = 0.91, p < 0.001) and *DELE1* (r = 0.98, R^2^ = 0.95, p < 0.001). On the other hand, among OXPHOS-related genes, *MT-COX1* showed a strong positive correlation with *MT-ND6* (r = 0.94, R^2^ = 0.89, p < 0.001), while *MT-ATP6* showed a strong positive correlation with Fpg-sensitive DNA damage (r = 0.70, R^2^ = 0.49, p < 0.01). Furthermore, *DNMT3a* showed a high positive correlation with *DELE1* (r ≈ = 1.00, R^2^ ≈ 0.99, p < 0.001) and *DNMT3b* (r = 0.85, R^2^ = 0.73, p < 0.001). Other high positive correlations include *miR-21* and *Fpg*-sensitive lesions (r = 0.69, R^2^ = 0.48, p < 0.01) as well as *miR-155* and *MT-ND6* (r = 0.36, R^2^ = 0.13, p < 0.05). High negative correlation includes *DRP1* and *MFN1* (r = −0.54, R^2^ = 0.29, p < 0.01); *MFN1* and miR-34a (r = −0.64, R^2^ = 0.41, p < 0.001), and *APE1* versus both *MT-COX1* and *MT-ND6* (r ≈ −0.42, R^2^ ≈ 0.18, p < 0.05). In addition, *miR-21* showed a negative correlation with *DNMT1* expression (r = −0.65, R^2^ = 0.42, p < 0.01).

### 3.4 Alteration in DNA methylation

The methylation analysis showed reduced methylation across all tested mitochondrial regions (D-loop1, D-loop2, 12s, and 16s) following administration of PS, PP, and PVC, compared with the control group without nanoparticle exposure. The reduction in methylation was more pronounced for PVC at 6 h, followed by PP and PS. For all mtDNA regions, prolonged exposure was unable to restore methylation (Supplementary Figure S11).

### 3.5 NPs exposure alters miRNA profiles and downstream target gene expression

PS, PP, and PVC led to significant time-related alterations in the expression of mitochondrial-associated miRNAs (Supplementary Figure S12). The three miRNAs: miR-21, miR-34a, and miR-155 exhibited an initial reduction in their expression up until 6 h, among which PVC was responsible for the highest reduction. Subsequently, the expression was sustained until 24 h, then increased until 48 h, with PVC showing the greatest increase in these miRNAs, followed by PS and PP (Supplementary Figure S13).

### 3.6 Evaluation of inflammatory cytokines

**FIGURE 5:**
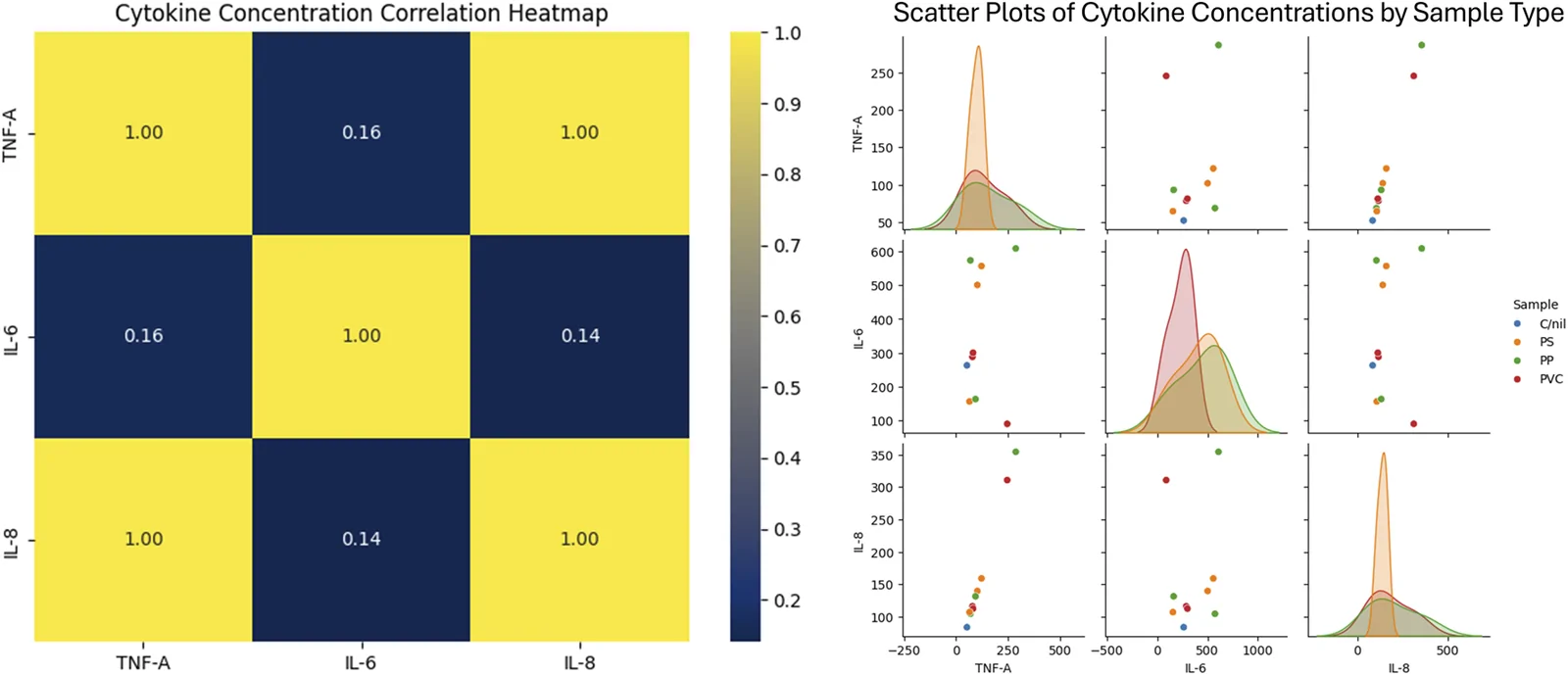
Combined analysis of TNF-α, IL-6, and IL-8 levels and their correlations. The left panel shows the Pearson correlation heatmap. TNF-α and IL-8 shows a positive correlation (r = 1.00), while correlations with IL-6 are low (TNF-α vs. IL-6: r = 0.16; IL-8 vs. IL-6: r = 0.14). The right panel shows pairwise scatter plots by group (control, PS, PP, PVC), with diagonal density plots indicating the distribution of each cytokine.

Exposure to the materials PS, PP, and PVC led to time- and material-dependent changes in cytokine production levels (Figure 5). IL-8 levels increased and remained elevated until 48 h, when the maximal response was observed. Elevated IL-8 expression was observed with PVC exposure (∼950 pg/mL), followed by PS and PP. The increase in TNF-α occurred as early as 6 h, peaked at 24 h in cells exposed to PP, and decreased by 48 h, almost returning to baseline in cells exposed to PVC. This suggests that the inflammation induced by PVC is transient, unlike the inflammatory response elicited by PS and PP. Additionally, IL-6 levels increased at 6 h, with PP exposure (∼580 pg/mL), followed by PS (∼500 pg/mL) and PVC (∼290 pg/mL). However, the highest level of IL-6 (∼610 pg/mL) was observed in PP-exposed cells at 24 h, followed by a decrease at 48 h. The correlation between TNF-α and IL-8 was very high (r = 1.00). On the other hand, IL-6 showed poor correlation with TNF-α (r = 0.16) and IL-8 (r = 0.14).

### 3.7 Measurement of mitochondrial electron chain complexes’ activity

**FIGURE 6:**
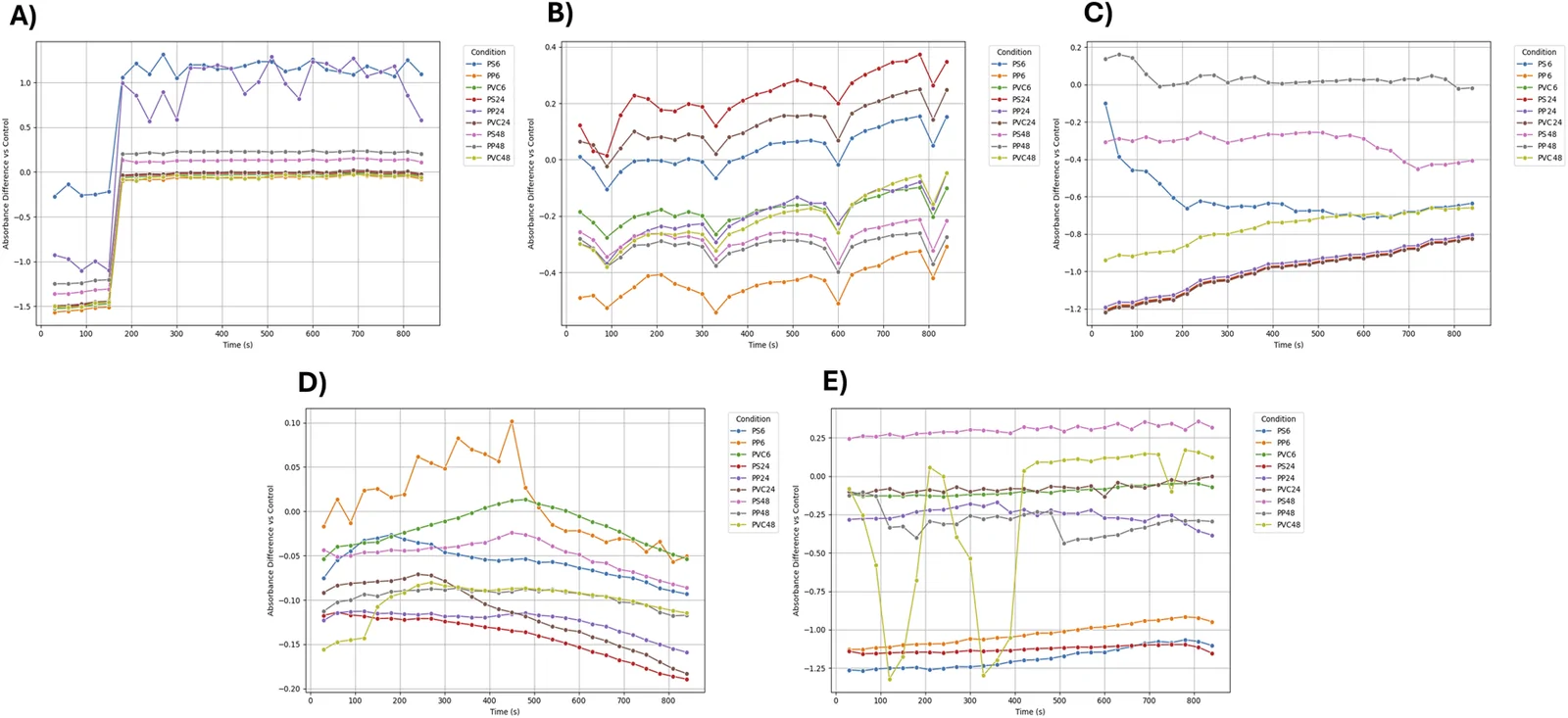
**(A–E)** Correspond to mitochondrial complexes I‐V respectively, each showing kinetic line plots of respiratory complex activity over time. The y-axis indicates absorbance difference relative to control, and the x‐axis represents time (s). Multiple lines within each plot represent different exposure conditions, including PS, PP, and PVC at 6, 24, and 48 h, allowing comparison of treatment‐dependent effects on individual mitochondrial complexes.

NPs exposure altered the activity of respiratory complexes I-V in a polymer- and time-dependent manner. Complex I activity decreased in all treated groups, with the notable reduction in PVC and PP treated cells, while PS treated cells showed less reduction (Figure 6A). Complex II showed an early increase, especially in PVC and PP treated cells, followed by a decrease (Figure 6B). Complex III remained mostly stable with small changes; PS and PP showed slightly higher activity at early time points, while PVC showed a gradual decrease (Figure 6C). Complex IV showed different patterns between polymers. PS and PP showed an early increase, while PVC showed a continuous decrease (Figure 6D). Complex V activity increased at early time points in all treated groups, especially in PS and PVC, but decreased later, with the strongest reduction in PVC (Figure 6E). Overall, these findings demonstrate that NPs disrupt OXPHOS in a progressive and polymer-dependent manner.

### 3.8 *In silico* molecular docking

**FIGURE 7:**
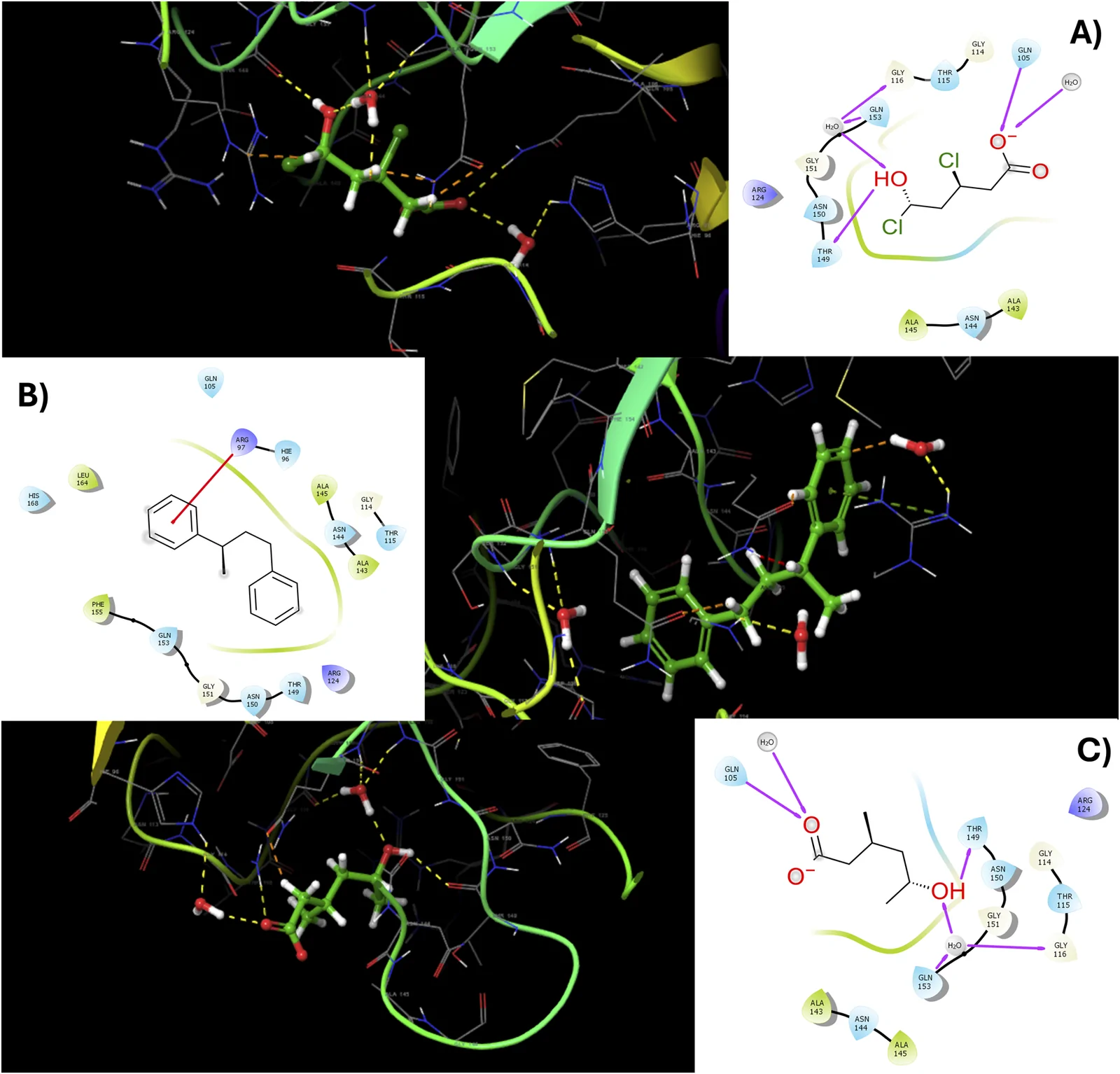
Molecular docking of NPs with mitochondrial complex I. **(A)** α-Carboxyl-ω-hydroxyl PVC bound to the Fe-S cluster with the highest affinity (−9.558 kcal/mol). **(B)** PS bound at the lipoyl-like cofactor site (−9.238 kcal/mol). **(C)** α-Carboxyl-ω-hydroxyl PP bound to the Fe-S cluster with a similar pattern.

Molecular docking analysis showed that oxidized NPs interact with mitochondrial Complex I in distinct ways, with predicted binding strengths ranging from −9.558 to −6.074 kcal mol^-1^. Docking simulations targeted several functionally important regions of Complex I, and the Fe–S cluster and lipoyl-like cofactor site were found to have the most favorable binding. Of all the ligands tested, the best predicted binding was observed for α-carboxyl-ω-hydroxyl poly (vinyl chloride) (PVC) with the Fe-S cluster (−9.558 kcal mol^-1^) (Figure 7A). Hydrogen bonding of the end groups, together with hydrophobic and van der Waals forces between the chlorinated hydrocarbon chain and the surrounding non-polar residues, contributed to the stability of the binding. PS also showed a strong binding affinity (−9.238 kcal mol^-1^) at the lipoyl-like cofactor site (Figure 7B). Its aromatic backbone fit into an aromatic-rich cavity and was stabilized mainly by hydrophobic and van der Waals interactions. Likewise, α-carboxyl-ω-hydroxyl poly (propylene) (PP) exhibited strong binding to the Fe-S cluster (−8.897 kcal mol^-1^) (Figure 7C). In this case, the oxidized terminal residues engaged in hydrogen bonding with surrounding polar residues, whereas the aliphatic core contributed to hydrophobic interactions. A hydrophobic surface analysis further supported that the polymer backbones fit well within hydrophobic binding cavities and that oxidized terminal groups help stabilize the ligands through hydrogen bonding (Supplementary Figure S14). In summary, this study demonstrates that the Fe-S cluster domain and lipoyl-like cofactor domain are effective binding sites for oxidized NPs, suggesting that these domains may affect the activity of Complex I (Table 1).

**TABLE 1:** Docking scores for oxidized NPs with complex I (5XTD).

NPs (ligand)	Docking score (kcal/mol)
At the flavin mononucleotide (FMN) binding site	
a-Carboxyl-ω-hydroxylpoly (styrene)	−7.99251
a-Aldehyde-ω-hydroxylpoly (styrene)	−6.75032
PS monomer terminal carboxyl	−6.66836
a-Carboxyl-ω-hydroxylpoly (vinyl chloride)	−6.15087
PVC MONOMER 2	−6.01624
At the (NADPH) binding site	
a-Carboxyl-ω-hydroxylpoly (styrene)	−6.07418
PS monomer terminal carboxyl	−5.48992
a-Carboxyl-ω-hydroxylpoly (propylene)	−5.47423
a-Aldehyde-ω-hydroxylpoly (styrene)	−5.35733
a-Carboxyl-ω-hydroxylpoly (vinyl chloride)	−5.2027
At the iron-sulfur cluster binding site	
a-Carboxyl-ω-hydroxylpoly (vinyl chloride)	−9.5581
a-Carboxyl-ω-hydroxylpoly (propylene)	−8.89698
PVC MONOMER 2	−7.21829
a-hydroxyl-ω-hydroxylpoly (vinyl chloride)	−4.23688
poly (styrene)	−4.17244
At the lipoyl-like cofactor binding site	
poly (styrene)	−9.23752

### 3.9 Predictive modeling of ND6 expression from complex I activity

**FIGURE 8:**
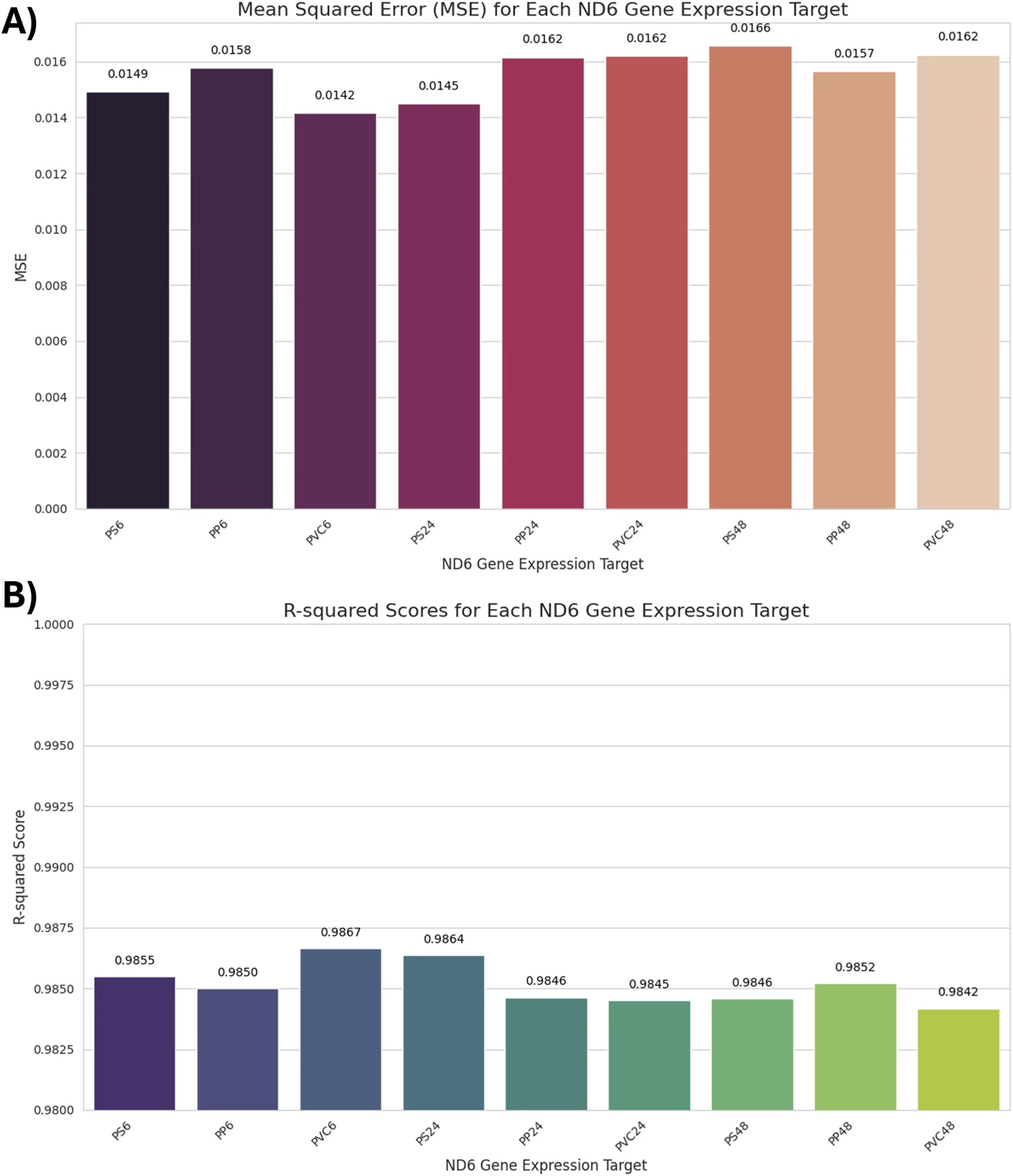
Performance of regression-based ND6 expression prediction. **(A)** R^2^ values for each ND6 target using a multi-output Random Forest model. **(B)** Mean squared error (MSE) values from the test dataset. Similar R^2^ values and low MSE across targets indicate stable performance.

**FIGURE 9:**
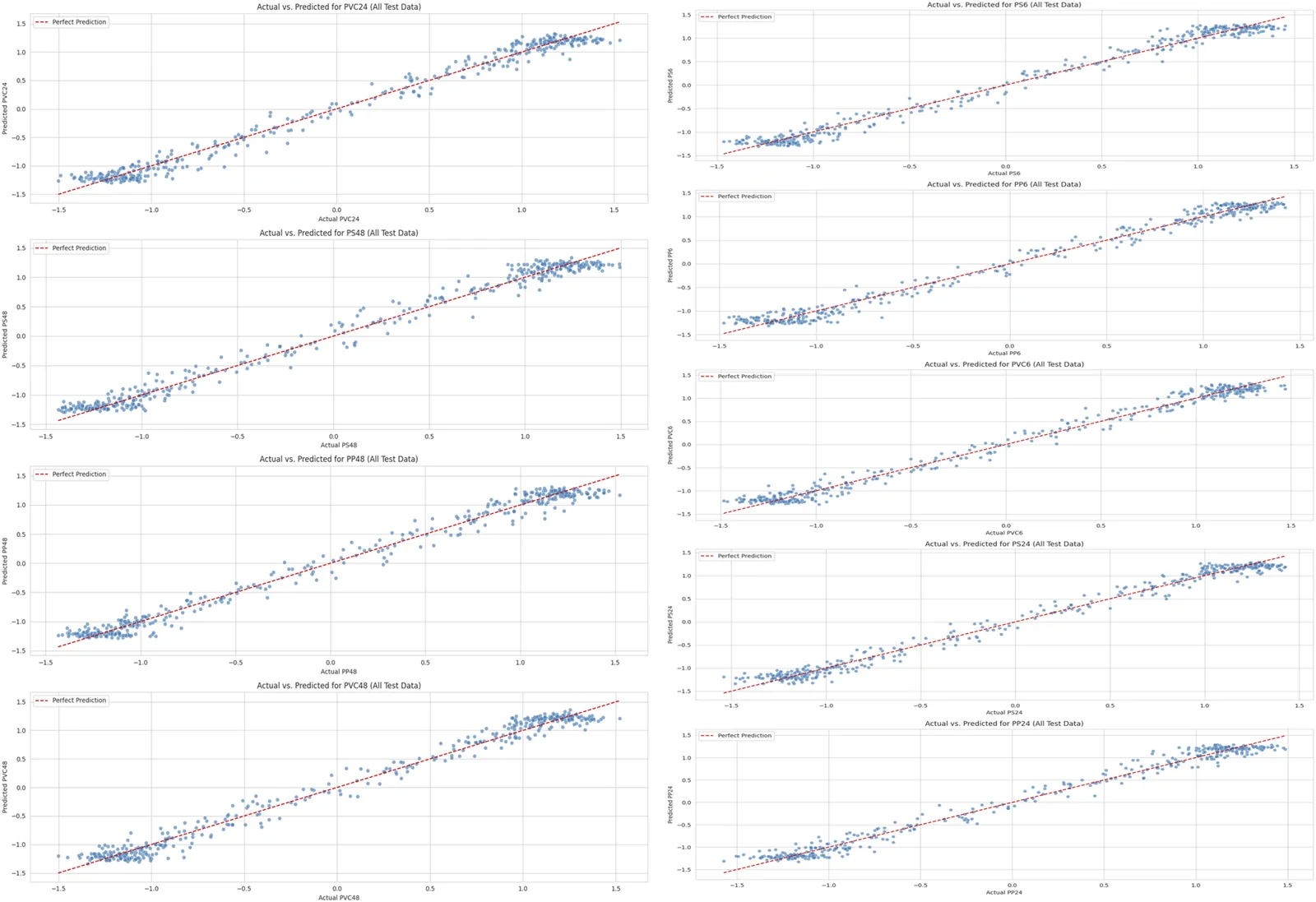
Residual analysis of ND6 gene expression predictions. The plot shows the residuals versus the predicted values, with the red dashed line indicating the absence of errors. There is an even distribution of points around zero with no pattern, indicating that the model’s bias and variance are minimal and that it fits the data well.

**FIGURE 10:**
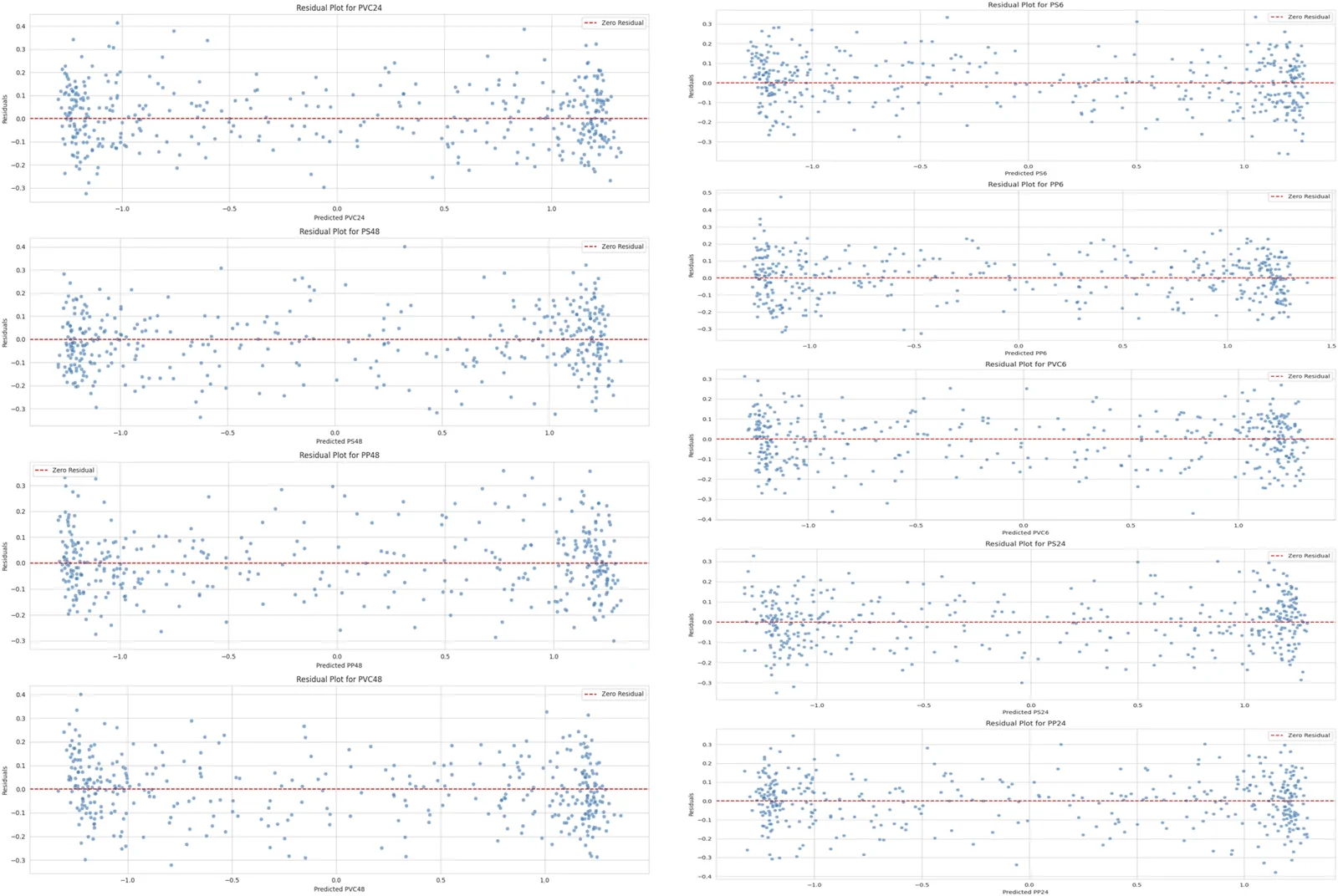
Observed vs. Predicted ND6 Gene expression by multi-output Random Forest Regression on test dataset. Plot of observed versus predicted ND6 Gene expression using multi-output Random Forest Regression.

The prediction model of ND6 gene expression based on complex I activity, using a multiple-output Random Forest regression, yielded satisfactory and reliable results on the independent test data set. There were high R^2^ values (0.984–0.987), indicating that more than 98% of the variation in ND6 expression is attributable to complex I. PVC6 and PS24 gave the best accuracy (Figure 8A). Also, the MSE values were low (0.014–0.017) for all the outputs (Figure 8B). There was no deviation in the residuals around zero (Figure 9). For the actual versus predicted graphs, the predictions matched the measurements in the entire range of the plot (Figure 10). This suggests that complex I activity corresponds with the measured ND6 expression.

## 4 Discussion

NPs have been identified as potential pollutants that can affect biological systems at multiple levels, including immune modulation, redox control, and mitochondrial activity (Lehner et al., 2019; Leslie et al., 2022). More recent findings have clearly established that intracellular NPs generate oxidative stress, mitochondrial damage, DNA damage, and inflammation across various cell models, reinforcing the critical importance of mitochondrial injury in the mechanism of nanoplastic toxicity (Casella and Ballaz, 2024; Tang et al., 2025). This study provides a comprehensive analysis of oxidative stress, mitochondrial bioenergetics, mitochondrial process kinetics, epigenetic modulation, inflammatory signaling, docking simulations, and regression modeling, focusing primarily on stress-response profiles and their subsequent cellular consequences. The concentrations used in this study were based on previous *in vitro* experimental work involving NPs, in which low doses (µg/mL), well below toxic levels, were used to mimic physiological internal exposures from various human routes of exposure to NPs (Stock et al., 2019). A dose of 1 μg/mL was chosen for the exposure based on two complementary considerations. Firstly, earlier *in vitro* studies have indicated that at this low dose, there is a measurable biological response without overt cytotoxicity, allowing the study of the mechanism of the cellular stress response (Stock et al., 2019). Secondly, current human biomonitoring data indicate that nanoparticle levels in circulation are within this range, suggesting that this level of exposure is environmentally plausible for mechanistic *in vitro* studies (Leslie et al., 2022). While the tissue accumulation of NPs may exceed circulating levels, the chosen exposure level is conservative for such studies. In addition, recent *in vitro* investigations of biological responses to NP exposure have also utilized 1 μg/mL as an exposure level to detect early biological changes without inducing cell death (Adamiak et al., 2025). Concentrations of 1 μg/mL in NPs are often used to assess nonlethal cellular stress responses, especially in continuously stimulated immune cells exposed to environmental particulates (Prata et al., 2020). Nevertheless, this concentration should not be interpreted as an exact equivalent of chronic human exposure, as biomonitoring levels reflect the cumulative effects of exposure to a variety of NPs, whereas this research addresses acute exposure to specific nanoplastics. The differences in exposure routes, the physical and chemical characteristics of particles, their distribution and accumulation, protein corona formation, the kinetics of clearance from the organism, and interindividual variations complicate such an extrapolation *per se*. This issue has recently been highlighted for the risk assessment of MPs and NPs, as well as for realistic human exposure to NPs (Cornelli et al., 2025; Song and Lee, 2026; Guo et al., 2026).

According to the physicochemical analysis, all examined polymers were nanometric in size. However, the PS polymer had the smallest hydrodynamic diameter among the polymers examined. All the tested NPs exhibited a low zeta potential. In other words, they exhibited minimal electrostatic repulsion. This aspect facilitates their interaction with biological membranes and proteins via hydrophobic and van der Waals forces. Thus, such interactions facilitate their incorporation into biological tissues as well as perturbation of biological membranes (Gurjar et al., 2024). Cellular uptake studies found that lymphocytic cells incorporated PS particles. It was confirmed using fluorescence microscopy and flow cytometry (Bhattacharjee et al., 2012). Currently, there is no information about the localization of PS nanoparticles within mitochondria (Zhang et al., 2024). Previous research has demonstrated the role of NPs in endolysosomal localization and mitochondrial impairment via redox imbalance and activation of inflammatory signaling pathways (Abulikemu et al., 2022). As a result, mitochondrial impairment observed during NP exposure should be considered the consequence of a cellular stress response rather than mitochondrial targeting by NPs. Despite additional useful information being obtainable via other physicochemical characterization techniques such as FTIR/Raman spectroscopy, electron microscopy, and time-dependent analysis of the colloidal stability of NPs in biological media, the main purpose of the current study was to compare the biological effects elicited by NPs produced from well-characterized commercial polymers. Thus, hydrodynamic size distribution and zeta potential were chosen as the main physicochemical parameters most closely related to the dispersion behavior of the nanoparticles. It should be noted that particle size and colloidal stability were not assessed during the 24–48 h incubation period because the dispersion of NPs in biological environments is dynamic and may vary due to agglomeration and protein corona formation. Future studies, including time-dependent physicochemical analyses of NPs under biological environmental conditions, will improve understanding of nanoplastic-cell interactions. Further, even though the nanoparticles were produced and studied using similar conditions, any variation in their hydrodynamic size, dispersion, or colloidal stability can account for the observed biological response. It is therefore recommended to interpret the current findings as comparative biological responses to identical exposure to NPs, rather than attributing them solely to the polymer type. Residual solvent contamination is a known methodological issue in solvent-based nanoplastic fabrication. While all nanoplastic suspensions were synthesized, prepared, and diluted using a standardized procedure prior to biological exposure, no direct measurement of residual solvents was conducted using analytical methods such as GC-MS. Further studies that include residual solvent measurements and vehicle control groups will provide additional insights into biological responses to NPs (Leslie et al., 2022).

NPs increased the levels of Fpg-sensitive purine oxidation products, especially when PP was used as the polymer. This process occurs quite rapidly and is a sign of ROS-induced oxidation of DNA bases, often serving as a marker of oxidative stress preceding activation of antioxidant protective mechanisms (Cadet et al., 2017; Li et al., 2023). The apparent decrease in the rate of repair responses can be explained by repair pathway saturation, inactivation of oxidative reactions, or cell death due to severe damage. Prolonged accumulation of ROS resulted from mtROS accumulation, mitochondrial permeability transition, and collapse of Δψm following exposure to PP nanoparticles. However, transient accumulation of mtROS was observed during PS and PVC exposure, followed by apoptosis (Xu et al., 2021; Yöntem and Ahbab, 2024). Oxidative stress can lead to genomic instability. Thus, the contribution of NPs in the induction of oxidative stress and genomic instability remains to be clarified. Prolonged accumulation of ROS, including mtROS, and a decrease in Δψm were observed at all periods. Oxidative stress is a known factor that can affect mitochondria, eliciting cellular responses that depend on its severity (Galluzzi et al., 2018).

Analysis of mitochondrial dynamics revealed the coincident upregulation of *DRP1* and *MFN1* expression, particularly during early PS exposure. Coordinated regulation of mitochondrial fission and fusion-associated proteins may be considered a stress-responsive mitochondrial adaptation that remodels mitochondria and optimizes bioenergetics in response to environmental challenges, consistent with previous research on environmentally stressed immune cells (Chang et al., 2023; Buck et al., 2015). Analysis of mitochondrial gene expression revealed a biphasic pattern in the regulation of OXPHOS genes encoded by mtDNA (*MT-ATP6, MT-COX1, and MT-ND6*). Early expression of the latter gene in response to NP exposure may be associated with the stress-induced transcriptional program of the mitochondrial stress response (Bennett et al., 2022). Mitochondrial metabolites are potent regulators of epigenetic enzymes; thus, NPs might affect inflammatory and metabolic homeostasis through their effect on mitogenetic regulation (Bhargava et al., 2019b). Increased expression of DNA repair markers was observed in all three treatment groups, with the greatest increase in *OGG1* after PS exposure and the greatest elevation in *APE1* mRNA levels after PVC exposure, indicating induction of the base excision repair pathway in response to oxidative DNA damage (Mishra et al., 2022b). It should be noted, however, that the induction of transcription does not necessarily indicate functional adaptation, especially under oxidative stress. Functional analysis suggests that Complex I is a stress-responsive mitochondrial component affected by NPs. Impairment of Complex I activity could inhibit electron transport from Complex I to Complex III, temporarily activating a compensatory mechanism involving activation of Complex II-mediated electron transport (Seward et al., 2025; Liu et al., 2026). The present situation falls under the category of an early mitochondrial stress response, which entails mitochondria temporarily sustaining ATP synthesis despite dysfunctional electron transport (Kaspar et al., 2021). As such, Complex I is considered a stress-responsive mitochondrion rather than a target of NPs.

Epigenetic profiling identified decreased expression of *DNMT1*, *DNMT3a*, and *DNMT3b*, along with putative hypomethylation in specific regions of the mitochondrial genome. mtDNA is highly vulnerable to oxidative base lesions, which could affect both methylation maintenance and epigenetic methylation profiling techniques (Poetsch, 2020; Nadalutti et al., 2022). Hypomethylation could therefore partially represent the effects of DNA lesions rather than genuine epigenetic reprogramming. The link between oxidative stress, defective repair, diminished DNMT levels, and altered mitochondrial gene expression supports the role of mitochondrial stress in epigenetic regulation (Dash et al., 2026). Although the current study clearly depicts an interplay among alterations in lncRNAs associated with mitochondria, DNA methyltransferase genes, and mtDNA methylation, it is not aimed at determining whether mitochondrial lncRNAs directly recruit DNMTs to the D-loop of mitochondria. However, recent studies have shown that the lncRNA encoded by the D-loop, lncMtDloop, regulates mitochondrial transcription by recruiting TFAM to mtDNA promoters, indicating the involvement of lncRNA-protein interactions in mitochondria. Although there is currently no direct evidence that mitochondrial lncRNAs recruit DNMTs to the D-loop, these findings provide a plausible basis for such mechanisms. The possibility of nanoplastic exposure-induced mitochondrial lncRNAs in DNMT recruitment leading to mtDNA methylation requires further investigation (Statello et al., 2020).

Upregulation of IL-6, TNF-α, and IL-8 gene expression can be observed in an inflammation profile test, possibly due to ROS signaling via the MAPK and NF-κB pathways (Bhargava et al., 2019a; Liu et al., 2020). In addition, increased IL-8 gene expression, but not IL-6, can be observed at a specific time point. Cytokine signaling associated with mitochondria has been reported, but exactly how this works at the molecular level remains to be explored. Additionally, the relationships among proteins involved in mitochondrial dynamics (DRP1 and MFN1), stress responses (OMA1 and DELE1), and epigenetics were noted. This reflects the presence of a concerted stress-response phenotype. Although the OMA1-DELE1 pathway has been associated with mitochondrial proteostatic stress in other models (Fessler et al., 2020), additional manipulation experiments will be required to establish this role in the current system.

The molecular docking technique was employed as an alternative method to investigate potential interactions between oxidized oligomers derived from NPs and components of mitochondrial respiratory chain complexes. While docking experiments alone cannot validate molecular interactions under physiological conditions, they may reveal binding sites important for the bioenergetic effects noted above (Hong et al., 2026). It is known that oxidized NP-derived oligomers can form hydrophobic interactions and hydrogen bonds with functional proteins, thereby affecting their conformation and function (Wang et al., 2023; Shi et al., 2024). The results obtained in our research demonstrated that, based on docking, oxidized oligomers might be located within the catalytically essential cavities of the Complex I holoenzyme (Domínguez-Ramírez et al., 2025). Nevertheless, certain questions regarding the formation of the protein corona, aggregation status, intracellular transport, and the accessibility of Complex I must be considered when interpreting the results. Likewise, recent research has underscored the importance of combining computational methods with experiments to gain a more holistic perspective on nanoplastic-protein interactions and their implications. Furthermore, the docking calculations have been conducted using oxidized polymer fragments rather than intact polymer chains. This implies that the predicted docking poses should be considered to provide information on interaction hot spots, and that the sterics of the polymer chains may not allow interaction with the internal binding sites under physiological conditions (Casella et al., 2025).

Consistent associations between Complex I activity and *MT-ND6* expression were observed regardless of polymer type or nanoparticle exposure duration. These results could be explained by the activation of bioenergetic and transcriptional stress responses, given that mitochondrial gene expression is regulated by oxidative balance and mtDNA stability (Khemraj et al., 2026). As mentioned above, regression analysis was conducted to identify correlations among the dependent variables; no causal relationships are claimed. For such determinations, high-resolution respirometry, specific interference with mitochondrial gene transcription, and subsequent protein validation are needed (Hafner et al., 2016). Some potential limitations must be kept in mind when analyzing the present data. First, PBMCs from several individuals were used in the experiments; thus, the study did not account for individual differences in mitochondrial and inflammatory responses. Second, changes in mitochondria and the epigenome have been analyzed at the transcriptional level and may not reflect actual protein levels and activities. Finally, molecular docking and regression analyses can be seen as auxiliary tools for exploratory analysis and should not be interpreted as evidence of a mechanism of action. It should be noted that particle aggregation, protein corona formation, intracellular localization, and chronic exposure conditions were not considered. Nevertheless, the obtained results form a basis for further investigations.

## 5 Conclusion

From the results of the current study, it can be concluded that the exposure of cells to the three NPs, namely, PS, PP, and PVC, in controlled laboratory conditions triggers distinct but similar mechanisms of cellular stress, such as oxidative stress, mitochondrial impairment, changes in the expression of genes related to the mitochondria, epigenetic changes, and inflammation. Although variations among polymer types have been identified, it is necessary to consider the composition and physicochemical properties of the materials studied. Among the analyzed mitochondrial indicators, respiratory chain Complex I appeared to be the most responsive stress mediator, whereas changes in *MT-ND6* expression levels were associated with alterations in mitochondrial function. Moreover, interrelationships among the mediators of the mitochondrial stress response, DNA repair mechanisms, epigenetic regulation, and inflammatory signaling suggest that NPs can affect multiple interconnected intracellular pathways. While these correlations can serve as clues to the possible pathways affected by nanoplastic exposure, the data presented cannot be considered evidence of the underlying mechanisms. Indeed, the docking and regression analysis were included in the present research merely for exploratory purposes and, hence, cannot confirm any interaction or correlation between molecules. Nonetheless, the combination of biochemical, molecular, computational, and bioenergetic analyses provides evidence that mitochondrial homeostasis is sensitive to nanoplastic-induced cellular stress. Overall, this work contributes to the growing body of knowledge on the effects of NPs on mitochondrial and epigenetic functions in immune cells. But understanding the physiological significance of these cellular responses would necessitate more studies, including proteomics, epigenomics, mitochondrial phenotypic analysis, environmentally relevant conditions for nanoplastic exposure, and animal modeling.

## Data Availability

The raw data supporting the conclusions of this article will be made available by the authors, without undue reservation.
